# Comparison of machine learning models and CEUS LI-RADS in differentiation of hepatic carcinoma and liver metastases in patients at risk of both hepatitis and extrahepatic malignancy

**DOI:** 10.1186/s40644-023-00573-8

**Published:** 2023-06-19

**Authors:** Jianming Li, Huarong Li, Fan Xiao, Ruiqi Liu, Yixu Chen, Menglong Xue, Jie Yu, Ping Liang

**Affiliations:** 1 Department of Interventional Ultrasound, Fifth Medical Center of Chinese PLA General Hospital, 100 West Fourth Ring Middle Road, Feng Tai District, Beijing, 100853 China; 2 Department of Ultrasound, Aero-space Center Hospital, Beijing, China; 3 Department of Ultrasound, Affiliated Hospital of Jilin Medical University, Changchun, China; 4 Department of Ultrasound, Chengdu Fifth People’s Hospital, Chengdu, China; 5 Department of Ultrasound, Guangxi Guigang People’s Hospital, Guigang, China

**Keywords:** Hepatocellular carcinoma, Liver metastasis, CEUS LI-RADS, Hepatitis

## Abstract

**Background:**

CEUS LI-RADS (Contrast Enhanced Ultrasound Liver Imaging Reporting and Data System) has good diagnostic efficacy for differentiating hepatic carcinoma (HCC) from solid malignant tumors. However, it can be problematic in patients with both chronic hepatitis B and extrahepatic primary malignancy. We explored the diagnostic performance of LI-RADS criteria and CEUS-based machine learning (ML) models in such patients.

**Methods:**

Consecutive patients with hepatitis and HCC or liver metastasis (LM) who were included in a multicenter liver cancer database between July 2017 and January 2022 were enrolled in this study. LI-RADS and enhancement features were assessed in a training cohort, and ML models were constructed using gradient boosting, random forest, and generalized linear models. The diagnostic performance of the ML models was compared with LI-RADS in a validation cohort of patients with both chronic hepatitis and extrahepatic malignancy.

**Results:**

The mild washout time was adjusted to 54 s from 60 s, increasing accuracy from 76.8 to 79.4%. Through feature screening, washout type II, rim enhancement and unclear border were identified as the top three predictor variables. Using LI-RADS to differentiate HCC from LM, the sensitivity, specificity, and AUC were 68.2%, 88.6%, and 0.784, respectively. In comparison, the random forest and generalized linear model both showed significantly higher sensitivity and accuracy than LI-RADS (0.83 vs. 0.784; all P < 0.001).

**Conclusions:**

Compared with LI-RADS, the random forest and generalized linear model had higher accuracy for differentiating HCC from LM in patients with chronic hepatitis B and extrahepatic malignancy.

**Supplementary Information:**

The online version contains supplementary material available at 10.1186/s40644-023-00573-8.

## Background

Hepatocellular carcinoma (HCC) is the sixth most common type of solid malignant tumor, with the liver also being the most common site of metastasis (70–97%) for extrahepatic tumors [1]. Accurate preoperative differentiation of liver metastasis (LM) from HCC using non-invasive tools is essential for deciding on management protocols, which include hepatectomy, liver transplantation, and systemic treatment [2–4]. Immune checkpoint inhibitors have been shown to be affective treatment options and have been approved for advanced HCC [5, 6]; however, systemic treatment of metastatic carcinoma needs to be determined according to the nature of the primary lesion [7]. For patients with both hepatic and extrahepatic malignancy, systemic treatment should be defined according to the primary or metastatic liver cancer. Sawatzki et al. demonstrated that contrast-enhanced ultrasound (CEUS) coupled with dynamic real-time imaging could provide higher temporal resolution and detect an additional 4% of LMs compared with conventional contrast-enhanced MRI [8].

The CEUS Liver Imaging Reporting and Data System (LI-RADS, 2017 version) was proposed as a standardized algorithm to diagnose liver cancer in patients at high risk because of disease such as hepatitis [9]. However, this system can be problematic in patients with chronic hepatitis and a history of primary extrahepatic malignancy, and who are at risk of both HCC and LM, because it can be difficult to distinguish between the two tumor types. A single retrospective study reported the diagnostic performance of MRI/CT LI-RADS in patients at risk of both HCC and LM [10], but because of sample size limitations, the authors did not further investigate the diagnostic features, nor methods to improve diagnostic performance. Zhou et al. reported that by adjusting early washout onset to 45 s in LI-RADS M, the specificity for differentiating HCC from intrahepatic cholangiocarcinoma could be significantly increased [11].

Nonetheless, there is no research confirming reliable criteria for the differential diagnosis of HCC from LM in patients with risks for both hepatitis and extrahepatic malignancy. Machine learning (ML) is an emerging field in medical image analysis, and it can be used to create accurate diagnostic models and identify complex relationships between variables and outcomes that may go undetected using traditional statistical approaches. Therefore, our multicenter retrospective study aimed to explore effective CEUS features and develop machine learning models for distinguishing HCC from LM in patients with chronic hepatitis and extrahepatic primary malignancy, and then to compare the performance of the models with that of LI-RADS.

## Methods

### Subjects

This retrospective multicenter study was conducted in accordance with the Declaration of Helsinki and ethics approval was obtained from all participating centers. The requirement for informed consent was waived because of the retrospective design. This study used patient data from a multicenter liver cancer database (http://www.usliver.org/home.html) and was registered at clinicaltrials.gov (NCT03871140). This study included 2811 patients with HCC and 399 with LM (recruited from 25 centers) who underwent CEUS with SonoVue (Bracco) between July 2017 and January 2022.

The inclusion criteria were: (i) the presence of chronic hepatitis B; (ii) the presence of visible nodules on CEUS; and (iii) confirmation of all visible nodules by postoperative pathology. The exclusion criteria were: (i) any treatment before CEUS; (ii) those that did not undergo CEUS at the corresponding center within three months before surgery; and (iii) absence of standard images available for review by the investigators. In patients with multiple tumors, the largest tumor was examined. All patient clinical information was collected from a database of electronic medical records.

### Contrast-enhanced ultrasound

CEUS was performed using different ultrasonography systems, including LOGIQ E9 (GE Healthcare), IU22 (Philips), and Aplio 500 (Canon Medical Systems) systems. All patients were examined after intravenous injection of the SonoVue ultrasound contrast agent, which contains sulfur hexafluoride encapsulated in a phospholipid shell. A 2.5-ml bolus injection was administered into the antecubital vein. The CEUS used a standardized protocol in all patients, with continuous assessment of the arterial phase (first 5 to 30–45 s) until maximum contrast enhancement was reached within the lesion, followed by intermittent scanning with short sweeps through the lesion at several time points as follows: (i) continuously performed CEUS for 2 min, (ii) followed by 5-s videos stored at intervals of 30 s until 5 min to evaluate the late venous phase. All ultrasound (US) images were stored in DICOM format and uploaded into the database. Video clips were reviewed on a computer screen by the investigators using a proprietary software package (Ebit Sanita, AET).

### LI-RADS algorithm

All liver tumors were categorized according to the 2017 CEUS LI-RADS criteria: LI-RADS-1 = definitely benign; LI-RADS-2 = probably benign; LI-RADS-3 = intermediate probability of malignancy; LI-RADS-4 = probably HCC; LI-RADS-5 = definitely HCC; LI-RADS-M = probably or definitely malignant, not necessarily HCC.

Two senior radiologists (Dr RQ. Liu and Dr YX. Chen, with > 10 years of experience in liver CEUS imaging) blinded to the pathological findings reviewed the CEUS images to reach a consensus for the evaluation of LI-RADS and CEUS features. One expert-level radiologist (Dr. F. Xiao, with > 15 years of experience in liver CEUS imaging) blinded to the pathological findings made the final diagnostic decision if no consensus was reached.

### Imaging analysis

To identify differences in CEUS features between HCC and LM, enhancement patterns were classified as follows: (1) arterial phase enhancement pattern, (2) homogeneity, (3) washout type, (4) unclear border, (5) tumor artery, (6) wheel enhancement, and (7) rim enhancement. Arterial phase enhancement pattern was defined as enhancement (hyper-, iso-, or hypo-enhancement) compared with the surrounding parenchyma over 10 to 30–45 s after administration. Homogeneity was defined as the same enhancement echoes in the arterial phase, whereas two or more enhancement echoes were categorized as heterogeneity. Washout type was defined according to the lesion becoming hypoechoic compared with the surrounding parenchyma in the portal-venous phase. Washout types included mild washout and marked washout, defined as washout within 60 s and markedly hypo-enhanced within two minutes, respectively. An unclear border was defined as nodular with a burr or fuzzy margin on all sides. A tumor artery was defined as obvious intratumor vasculature in the arterial phase. Wheel and rim enhancement were defined as wheel and rim type enhancement in the arterial phase (Table S1). In addition, the optimal mild washout time was explored to maximize diagnostic performance.

### Machine-learning model development

ML-based algorithms were used to develop a predictive model to find useful independent predictors of the outcome under investigation. Three ML-based algorithms were evaluated: (1) a gradient-boosted model (GBM) [12], (2) a random forest model [13], and (3) a generalized linear model (GLM) [14]. Hyperparameter tuning of the ML algorithms was performed on selected CEUS feature lists using a grid search with 5-fold cross-validation with ten repeats. The above algorithms were used to analyze the contribution of each imaging feature (gain) to the rates of LM. After identifying the most appropriate imaging features, we used these as predictor variables to construct corresponding ML models.

### Online model deployment

After training, the GLM was saved in a file that could be loaded online. To make it available, we created a web application that can make predictions from new data entered by the user. Using the user’s answers to five questions, the application provides the probability of HCC/LM diagnosis.

### Statistical analyses

In the baseline comparisons, Student’s *t*-test or the Mann–Whitney U test was used to compare continuous variables, and Pearson’s chi-square or Fisher’s exact test was used to evaluate categorical variables. Propensity-score models were calculated using a multivariable logistic regression model. All patients included in this study had hepatitis, and the presumed confounders (age, diameter, sex, abdomen pain, vascular invasion, and extrahepatic tumor) were used as independent variables for the model fitting for the training and validation cohorts. Propensity score matching was used to reduce the influence of confounders and selection bias. The enrolled patients were matched using 1:1 nearest neighbor matching with a caliper distance set at 0.05 standard deviations of the logit of the propensity score for the training cohort and validation cohort. The training cohort consisted of patients with hepatitis. The validation cohort consisted of patients with both hepatitis and extrahepatic tumor.

**Kappa coefficients were used to assess interobserver consistency in LI-RADS and arterial phase enhancement patterns between two senior radiologists. A restricted cubic spline fitting was used to visualize the nonlinear relationship between the independent variable and the dependent variable** [15, 16]. A two-piece-wise linear regression model using a smoothing function was used to examine the effect of different thresholds for the mild washout time on LM rates. The threshold level was determined using trial and error, and included selecting the point in the function curve showing a sharp change and a pre-defined interval on either side, and then choosing the point that gave the maximum model likelihood.

The relationships between CEUS features of LM (in the form of rank variables, continuous variables, and other variables) were assessed using Pearson correlation coefficients. CEUS features showing significant associations with HCC and LM were screened using a GLM with the Hosmer–Lemeshow goodness-of-fit set to the confidence interval (CI) for exp(B), followed by the designation of dummy variables for multi-categorical variables and backward regressions to further screen variables. For GBM and the random forest model, backward stepwise analysis was used to select the variables according to the Akaike information criterion.

Pathological results were used as the reference standard, and optimal cutoff values for the prediction of LM were identified using the highest Youden index and maximization of sensitivity and specificity. The GBM, random forest, and GLM were run using the gbm R package, randomForest R package, and glm R package. Two-sided P-values < 0.05 were considered statistically significant. All data were analyzed with R software version 4.1.0 (http://www.r-project.org).

## Results

### Patient characteristics

**Fig. 1:**
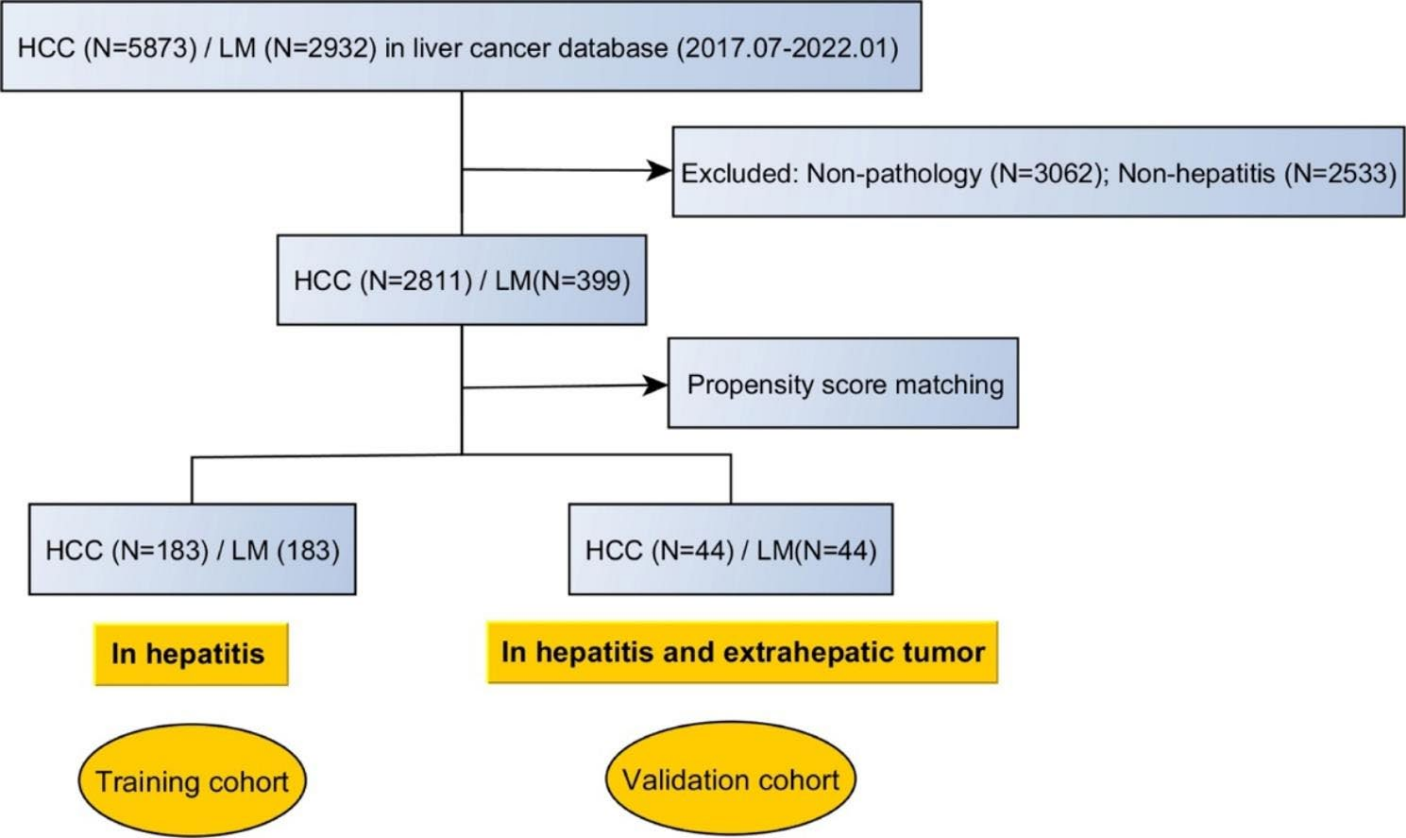
A flowchart shows patients’ inclusion and research design

A total of 3210 patients were included in the multicenter database (Fig. 1). After PSM, 366 patients with hepatitis were selected as the training cohort, and 88 patients with both hepatitis and extrahepatic primary malignancy were selected as the external validation cohort. There were no significant differences in clinical characteristics between patients with HCC and LM in the training and validation cohorts (Table 1).

**Table 1:** Patient characteristics

Parameter	All data			Training Cohort			Validation Cohort		
	HCC(N = 2811)	LM(N = 399)	*P*	HCC(N = 183)	LM(N = 183)	*P*	HCC(N = 44)	LM(N = 44)	*P*
**Age**	57.39 ± 11.39	58.79 ± 10.61	0.020	57.26 ± 11.16	59.15 ± 9.67	0.083	61.52 ± 10.84	59.75 ± 11.27	0.454
**Diameter**	4.99 ± 3.32	4.43 ± 2.81	0.001	5.36 ± 11.93	5.15 ± 4.82	0.821	4.97 ± 4.23	5.03 ± 5.82	0.954
**Sex**			< 0.001			0.348			0.280
Female	504 (17.93%)	154 (38.60%)		46 (25.14%)	54 (29.51%)		16 (36.36%)	21 (47.73%)	
Male	2307 (82.07%)	245 (61.40%)		137 (74.86%)	129 (70.49%)		28 (63.64%)	23 (52.27%)	
**Jaundice**			0.096			0.410			0.148
No	2737 (97.37%)	394 (98.75%)		179 (97.81%)	181 (98.91%)		41 (95.35%)	44 (100.00%)	
Yes	74 (2.63%)	5 (1.25%)		4 (2.19%)	2 (1.09%)		2 (4.65%)	0 (0.00%)	
**Abdomen pain**			0.001			0.068			0.141
No	2354 (83.74%)	308 (77.19%)		153 (83.61%)	139 (75.96%)		36 (83.72%)	31 (70.45%)	
Yes	457 (16.26%)	91 (22.81%)		30 (16.39%)	44 (24.04%)		7 (16.28%)	13 (29.55%)	
**Vascular invasion**			0.010			0.091			0.091
No	2572 (91.50%)	380 (95.24%)		167 (91.26%)	175 (95.63%)		39 (88.64%)	43 (97.73%)	
Yes	239 (8.50%)	19 (4.76%)		16 (8.74%)	8 (4.37%)		5 (11.36%)	1 (2.27%)	
**Multiple tumors**			0.993			0.224			0.080
No	2262 (80.47%)	321 (80.45%)		154 (84.15%)	145 (79.23%)		40 (90.91%)	41 (93.18%)	
Yes	549 (19.53%)	78 (19.55%)		29 (15.85%)	38 (20.77%)		4 (9.09%)	3 (6.82%)	
**Extrahepatic tumor**			< 0.001			NA			NA
No	2691 (95.73%)	7 (1.75%)		183 (100%)	0 (0.00%)		0 (0.00%)	0 (0.00%)	
Yes	120 (4.27%)	392 (98.25%)		0 (0.00%)	183 (100%)		44 (100.00%)	44 (100.00%)	
**Chronic hepatitis B**			0.706			NA			NA
No	0 (0.00%)	0 (0.00%)		0 (0.00%)	0 (0.00%)		0 (0.00%)	0 (0.00%)	
Yes	2811(100%)	399 (100%)		183 (100%)	183 (100%)		44 (100.00%)	44 (100.00%)	

LM: Liver metastasis; HCC: Hepatocellular carcinoma; NA: Not available

### Interobserver agreement

We found good interobserver agreement between the two senior radiologists with respect to both LIRADS and arterial phase enhancement patterns (k = 0.845 and k = 0.850, respectively; Tables S2 and S3).

### Exploration of the optimal mild washout time for differentiating between HCC and LM

**Fig. 2:**
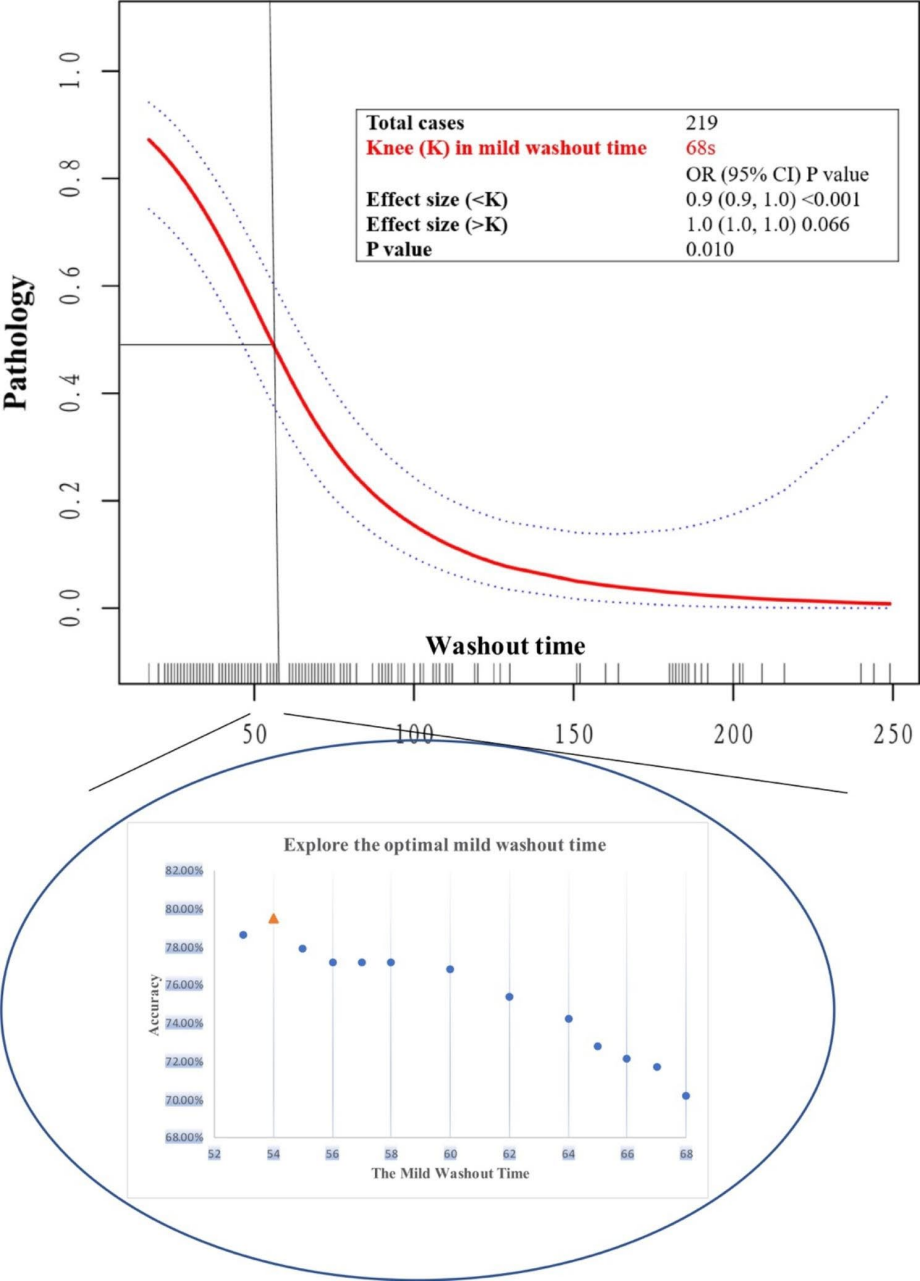
Explore the knee of the optimal mild washout time The rate of a composite LM outcome was plotted against the mild washout time and fitted with a curve indicating the relationship between the washout time and the rate of LM Abbreviations: LM, Liver metastasis

There was a nonlinear relationship between a mild washout time and the risk of LM. The risk of LM was significantly lower (P < 0.001) with a mild washout time up to an abrupt change point (mild washout time = 68 s), with the odds ratio (OR) of the mild washout time being 0.9 (95% CI: 0.9–1.0). With a mild washout time > 68 s, the relationship between the risk of LM and mild washout time was not significant (P = 0.066). Comparison of the mild washout time (53–68 s) between HCC and LM revealed that a washout time of 54 s was the best point for differentiating between them, with a diagnostic accuracy of 79.4% (Table S4; Fig. 2). Therefore, we created a washout type II feature, which combined mild washout (≤ 54 s) and marked washout into a predictor variable for LM.

### Univariate and correlation analysis of CEUS features for differentiating between HCC and LM

**Fig. 3:**
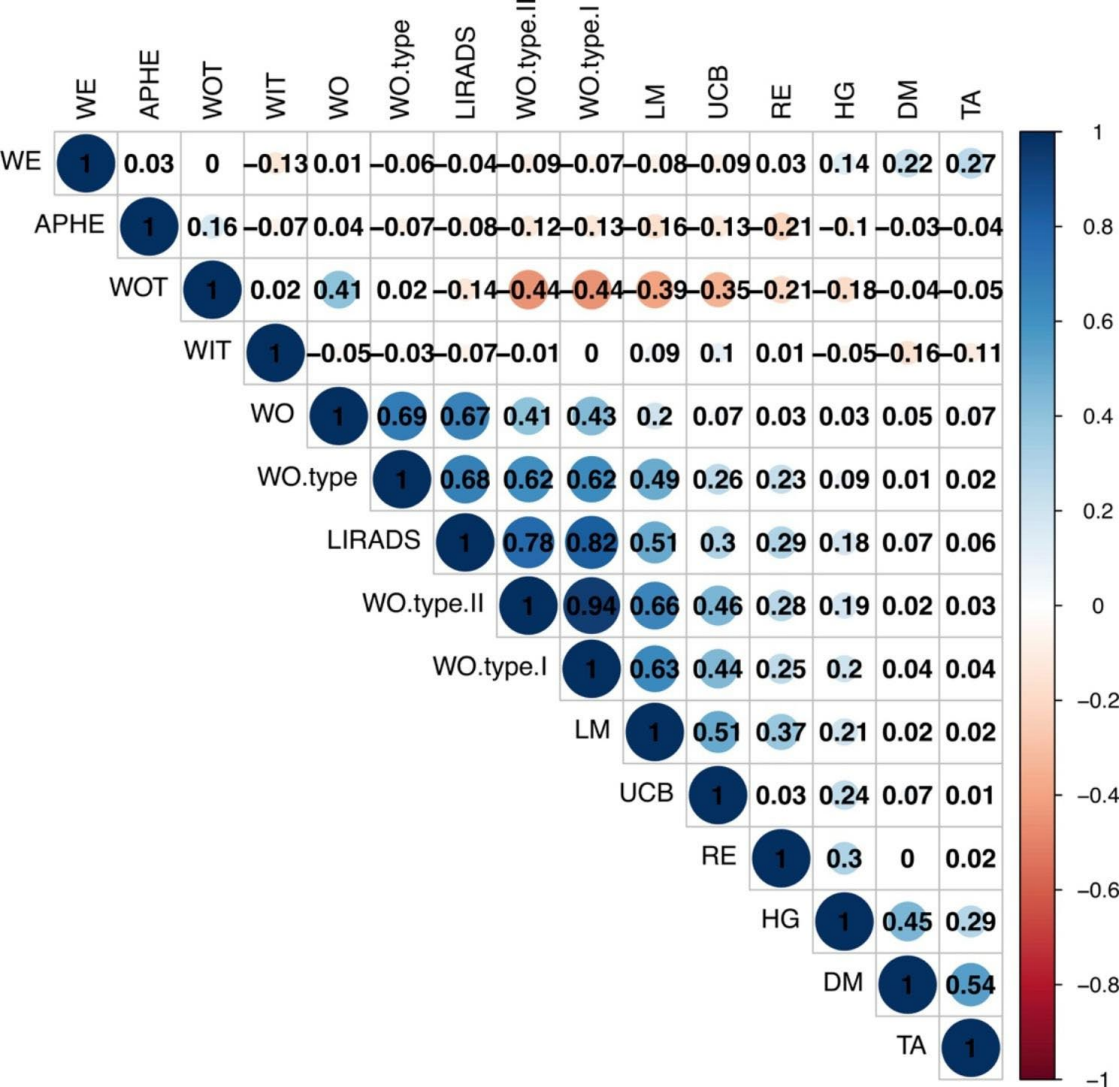
Univariate correlation matrix for the different CEUS features

According to the 2017 CEUS LI-RADS criteria applied to the training cohort, 138 HCCs and 18 LMs were evaluated as LI-RADS 3–5, and 45 HCCs and 165 LMs were evaluated as LI-RADS-M. Compared with HCCs, LMs showed significantly higher proportions of arterial hypo-enhancement (26.8%), heterogeneity (60.7%), washout (94.5%), washout type I (88%), washout type II (86.3%), unclear border (79.2%), and rim enhancement (27.9%) (all P < 0.001). In the univariate analysis, arterial phase enhancement, heterogeneity, washout times, washout, washout type, washout type I, washout type II, unclear border, rim enhancement, and LI-RADS classification were significant risk factors for LM (P < 0.001). Correlation analysis showed that washout times, washout type, washout type I, washout type II, unclear border, rim enhancement and LI-RADS classification were correlated with LM (3<|r|) (Table 2; Fig. 3).

**Table 2:** CEUS features in the training and validation cohort

Parameter	Correlation index (r)	Training Cohort			Validation Cohort		
		HCC (N = 188)	LM (N = 188)	*P*	HCC (N = 44)	LM (N = 44)	*P*
**Diameter**	0.01738	4.29 ± 2.76	4.46 ± 2.77	0.343*	4.23 ± 2.93	4.01 ± 2.31	0.822*
**Wash-in Time**	0.08857	14.59 ± 4.78	15.37 ± 5.87	0.209*	13.70 ± 3.78	15.32 ± 6.03	0.323*
**Wash-out Time**	-0.39343	75.85 ± 63.32	35.26 ± 20.82	< 0.001*	70.66 ± 60.49	34.41 ± 16.69	< 0.001*
**Arterial phase enhancement**	-0.16467			< 0.001			< 0.001*
Hyper-enhancement		165 (90.16%)	114 (62.30%)		40 (90.91%)	26 (59.09%)	
Iso-enhancement		9 (4.92%)	20 (10.93%)		3 (6.82%)	6 (13.64%)	
Hypo-enhancement		9 (4.92%)	49 (26.78%)		1 (2.27%)	12 (27.27%)	
Homogeneity	0.20708			< 0.001			0.019
Homogeneity		108 (59.02%)	72 (39.34%)		27 (61.36%)	16 (36.36%)	
Heterogeneity		75 (40.98%)	111 (60.66%)		17 (38.64%)	28 (63.64%)	
**Washout**	0.20094			< 0.001			0.179
No		36 (19.67%)	10 (5.46%)		7 (15.91%)	3 (6.82%)	
Yes		147 (80.33%)	173 (94.54%)		37 (84.09%)	41 (93.18%)	
**Washout type**	0.49244			< 0.001			< 0.001
No		37 (20.22%)	11 (6.01%)		8 (18.18%)	3 (6.82%)	
Mild washout		141 (77.05%)	78 (42.62%)		33 (75.00%)	20 (45.45%)	
Marked washout		5 (2.73%)	94 (51.37%)		3 (6.82%)	21 (47.73%)	
**Washout type I**	0.63146			< 0.001			< 0.001
No/Mild washout (> 60s)		139 (75.96%)	22 (12.02%)		30 (68.18%)	5 (11.36%)	
Marked/Mild washout (≤ 60s)		44 (24.04%)	161 (87.98%)		14 (31.82%)	39 (88.64%)	
**Washout type II**	0.65846			< 0.001			< 0.001
No/Mild washout (> 54s)		144 (78.69%)	25 (13.66%)		35 (79.55%)	5 (11.36%)	
Marked/Mild washout (≤ 54s)		39 (21.31%)	158 (86.34%)		9 (20.45%)	39 (88.64%)	
**Unclear Border**	0.50787			< 0.001			< 0.001
No		134 (73.22%)	38 (20.77%)		29 (65.91%)	10 (22.73%)	
Yes		49 (26.78%)	145 (79.23%)		15 (34.09%)	34 (77.27%)	
**Tumor artery**	0.02126			0.619			1.000
No		143 (78.14%)	139 (75.96%)		36 (81.82%)	36 (81.82%)	
Yes		40 (21.86%)	44 (24.04%)		8 (18.18%)	8 (18.18%)	
**Wheel enhancement**	-0.07901			0.121*			1.000*
No		177 (96.72%)	182 (99.45%)		43 (97.73%)	43 (97.73%)	
Yes		6 (3.28%)	1 (0.55%)		1 (2.27%)	1 (2.27%)	
**Rim enhancement**	0.37034			< 0.001			< 0.001
No		179 (97.81%)	132 (72.13%)		44 (100.00%)	31 (70.45%)	
Yes		4 (2.19%)	51 (27.87%)		0 (0.00%)	13 (29.55%)	
**LI-RADS**	0.50722			< 0.001			< 0.001
3		6 (3.28%)	4 (2.19%)		0 (0.00%)	2 (4.55%)	
4		27 (14.75%)	3 (1.64%)		5 (11.36%)	1 (2.27%)	
5		105 (57.38%)	11 (6.01%)		25 (56.82%)	2 (4.55%)	
M		45 (24.59%)	165 (90.16%)		14 (31.82%)	39 (88.64%)	

LM: Liver metastasis; HCC: Hepatocellular carcinoma. Correlation index (r) is Pearson’s correlation coefficient. P^*^: Continuous variables—Kruskal Wallis rank sum test; Count variables < 10—Fisher exact test

The correlation index is Pearson’s correlation coefficient. The size of the circle reflects the degree of statistical significance

Abbreviations: DM, Diameter; WIT, Wash-in Time; WOT, Wash-out Time; APHE, Arterial phase enhancement; HG, Homogeneity; WO, Washout; WO type, Washout type; WO type I, Washout type I; WO type II, Washout type II; UCB, Unclear Border; TA, Tumor artery; WE, Wheel enhancement; RE, Rim enhancement; LM, Liver metastasis

### CEUS feature selection

**Fig. 4:**
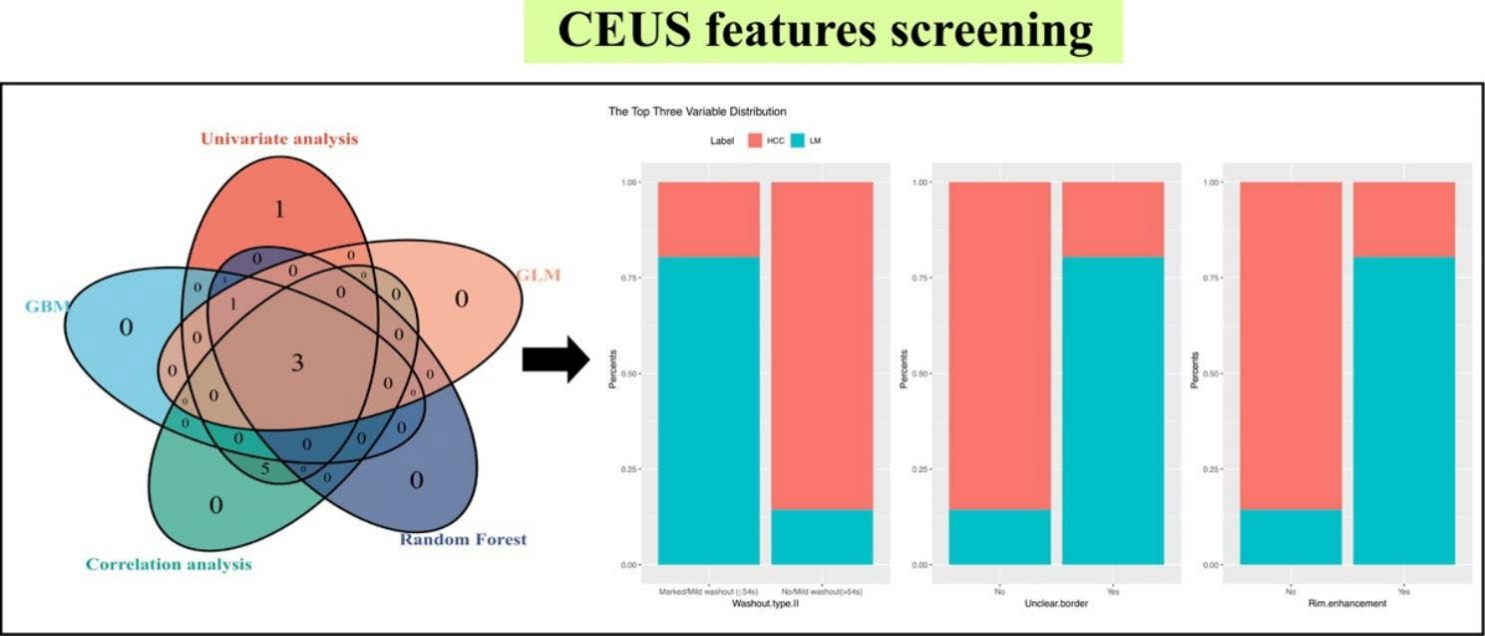
CEUS variables screening by univariate, correlation analysis and machine learning models

After the washout time was optimized, univariate analysis and correlation analysis were performed between features of LM and HCC. The selected CEUS feature list included five variables: arterial phase enhancement, homogeneity, washout type II, unclear border, and rim enhancement, according to the above analysis and expert advice. We generated the GBM, random forest, and GLM using the above-selected features, taking pathological results as the reference standard diagnoses. The three most influential predictors in the ML models applied to the training cohort were washout type II, unclear border, and rim enhancement (Fig. 4).

### Comparison of machine learning models with LI-RADS

**Fig. 5:**
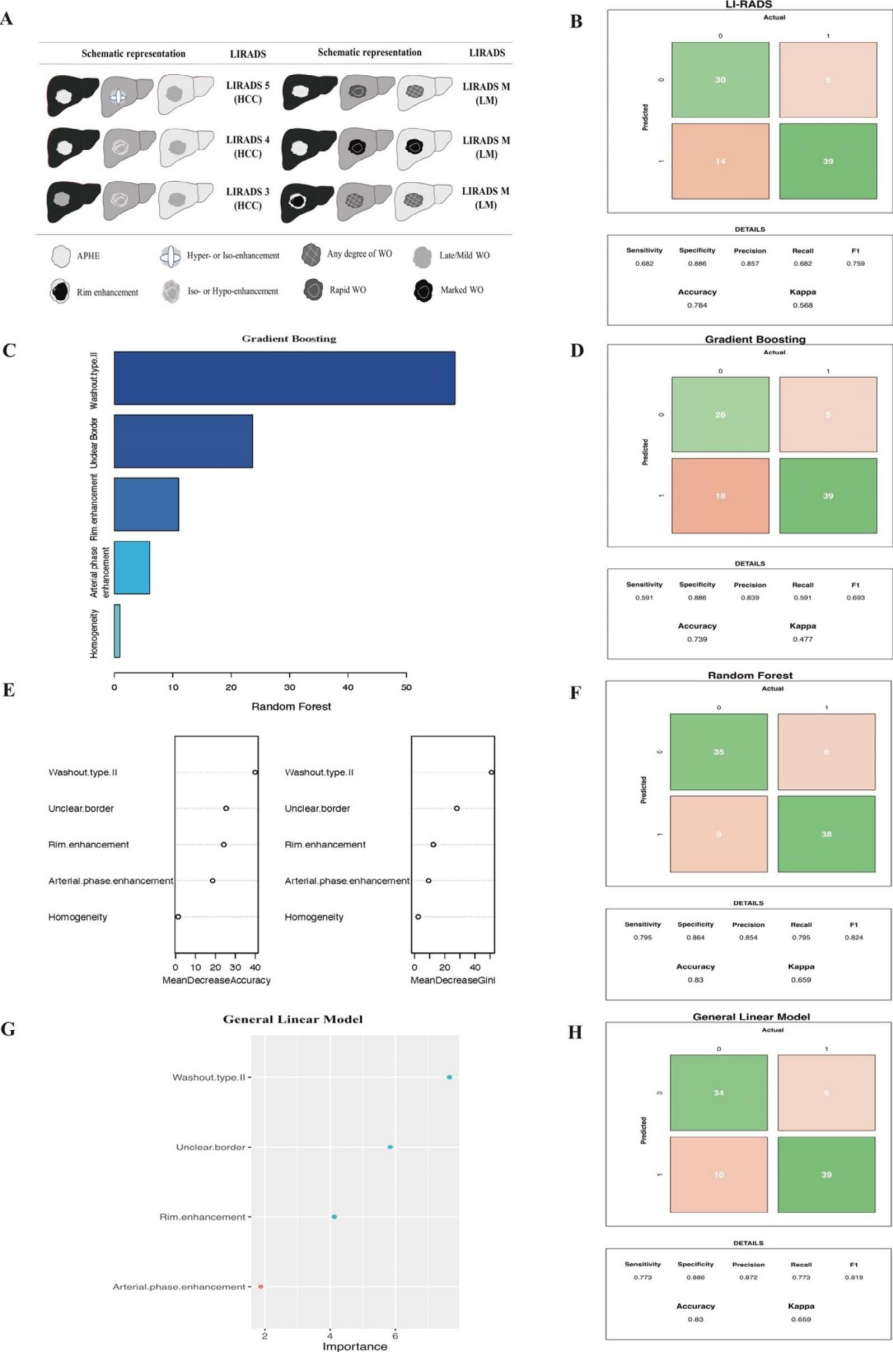
Importance of the predictor variables and the diagnostic performance of LI-RADS and machine learning models (**A**). Schematic representation in the CEUS LI-RADS; (**B**) The diagnostic performance of LI-RADS; (**C**). Variables show in the Gradient Boosting Model; (**D**). The diagnostic performance in the Gradient Boosting Model; (**E**). Variables show in the Random Forest; (**F**). The diagnostic performance in the Random Forest; (**G**). Variables show in the General Linear Model; (**H**). The diagnostic performance in the General Linear Model

We compared the diagnostic performance of LI-RADS, GBM, random forest, and the GLM on the external validation dataset consisting of patients with double risks of LM and HCC. The sensitivity, specificity, positive predictive value (PPV), negative predictive value (NPV), and accuracy of LI-RADS were 68.2%, 88.6%, 85.7%, 26.4%, and 0.784, respectively. The GBM’s sensitivity, specificity, PPV, NPV, and accuracy were 75.0%, 86.4%, 83.9%, 28.1%, and 0.807, respectively, with sensitivity being significantly higher than that of LI-RADS. The sensitivity, specificity, PPV, NPV, and accuracy were 79.5%, 86.4%, 85.4%, 19.1%, and 0.83, respectively, for the random forest, and 77.3%, 88.6%, 87.2%, 20.4%, and 0.83 for the GLM. The random forest and GLM showed significantly higher sensitivity and accuracy than LI-RADS (P < 0.001; Table 3; Fig. 5).

**Table 3:** Comparison of diagnostic performance of the different criteria for HCC/LM in the validation cohorts

Diagnostic criteria	Sensitivity	Specificity	PPV	NPV	Accuracy
**LI-RADS**	68.2%	88.6%	85.7%	26.4%	0.784
**Gradient Boosting Model**	75.0%*	86.4%	83.9%	28.1%	0.807
**Random Forest**	79.5%*	86.4%	85.4%	19.1%*	0.830*
**General Linear Model**	77.3%*	88.6%	87.2%	20.4%*	0.830*

Machine Learning (GBM, RF and GLM): based on Arterial phase enhancement, Homogeneity, Washout type II, Unclearly border and Rim enhancement

GBM: Gradient Boosting Model; RF: Random Forest; GLM: General Linear Model

^*^There was statistical difference compared with LI-RADS (Two-sided P-values < 0.05)

### Model interpretation at the individual scale

**Fig. 6:**
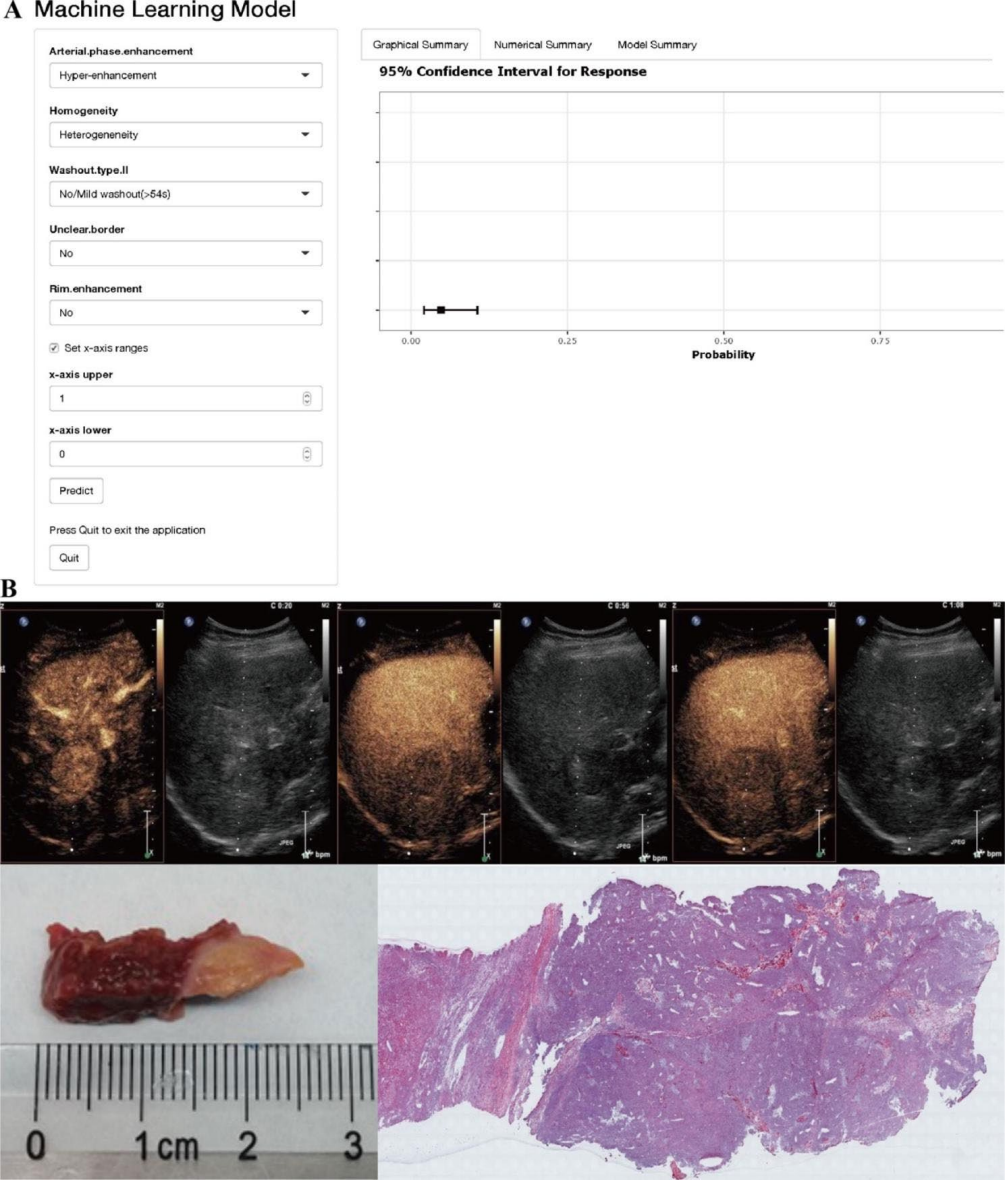
Model interpretation at the actual case (**A**). Machine learning online model deployment. (**B**). One case shows arterial phase hyper-enhancement nodule, clear border, and mild washout at 56s, then the postoperative pathology confirmed HCC.

The GLM model is deployed online at the following URL: (https://livercancer.shinyapps.io/DynNomapp/). The user answers five questions to obtain a prediction of the probability of HCC/LM (Fig. 6A). As an example, we provide the features and prediction for one patient with a liver tumor. This patient was highly suspected of HCC or LM because of a history of chronic hepatitis and colorectal cancer, and biopsy was hard to perform because of a high-risk location that was adjacent to a blood vessel. The lesion showed arterial phase hyperenhancement, heterogeneity, a clear border, a mild washout time of 56 s, and non-rim enhancement on CEUS. The observation was categorized as LM according to the LI-RADS algorithm, whereas to the contrary, the GLM evaluated it as HCC. After discussion at the multidisciplinary team meeting and according to the patient’s choice, this patient underwent hepatectomy instead of systemic treatment. The postoperative pathology confirmed HCC (Fig. 6B).

## Discussion

When patients have both chronic liver disease and a history of extrahepatic primary malignancy, and are at risk of both HCC and LM, the risk of misdiagnosing metastasis as HCC on imaging could be higher than in patients without a history of extrahepatic malignancy [17, 18]. In this study, we evaluated the effectiveness of CEUS features and established ML models for HCC and LM. We observed marked differences in the enhancement features of vascular characteristics and tumor morphology between HCC and LM. We then developed an ML model using the training cohort and subsequently validated it using an independent cohort of patients with hepatitis and extrahepatic tumors [19].

Terz et al. reported that LI-RADS could result in a reliable non-invasive diagnosis in patients with HCC [20]. In the present study, LI-RADS showed moderate diagnostic performance (accuracy: 0.784) in the differentiation between HCC and LM in high-risk patients. Univariate and correlation analysis revealed that LI-RADS moderately correlated with LM and HCC. This suggests that the LI-RADS algorithm could contribute to the identification of patients with HCC and LM.

A finding of note is that while rim enhancement is applied in the LI-RADS algorithm [21, 22], we found that washout type II and unclear borders were significantly correlated with LM. In the training cohort, LM showed a significantly higher proportion of unclear borders (145/183, 79.2%) than HCC (49/183, 26.8%). Using the tumor margins as the diagnostic characteristic allowed differentiation between HCC and LM. Higher proportions of unclear borders in LM might be due to more infiltration of surrounding tissues by LM in comparison with HCC [23], with HCC often having a pseudo capsule composed of inflamed and fibrotic tissue, especially in cirrhotic livers [24]. Therefore, LM shows a more unclear border than HCC on CEUS.

In terms of washout patterns, early mild washout and marked washout were more frequently detected in LM (161/183 cases, 88.0%) than in HCC (44/183 cases, 24.0%), which is consistent with previous research [11, 25]. We found that early mild washout onset tended to be ≤ 54 s in most LMs, rather than the < 60 s state in LI-RADS. Mild washout (≤ 54 s) and marked washout were independent diagnostic indicators for differentiating between HCC and LM, and we therefore integrated mild washout (≤ 54 s) and marked washout into the washout type II feature, which was selected as one of the top three features for the ML models.

**In this study, we investigated the most effective CEUS features for differentiating HCC and LM.** By identifying and utilizing the variables washout type II, unclear border, and rim enhancement, we incorporated expert advice and added two potentially valuable features (arterial phase enhancement and homogeneity) to the ML models. With respect to the ML models, GBM is a machine learning technique used in regression and classification tasks, and is well known for converting weak learners into strong learners [26]. **Good performance in a learning algorithm is critical to developing an accurate diagnostic model, and** our GBM had significantly higher sensitivity than LI-RADS. We were surprised that the random forest and GLM also showed significantly higher sensitivity and accuracy than LI-RADS.

Particular strengths of our study are that we tried to acknowledge the potential value of LI-RADS in differentiating between HCC and LM while building ML models to improve diagnostic performance in an independent cohort with both hepatitis and extrahepatic tumor. Our findings could provide an additional diagnostic reference for HCC and LM.

Our research is subject to some limitations. First, only HCC and LM were included in this study, with the exclusion of other hepatic malignancies, resulting in high specificity (88.6%) for LI-RADS. For this reason, we developed an effective CEUS algorithm for differentiating HCC from LM in high-risk patients. Second, we did not directly compare the diagnostic performance of LI-RADS and ML models in the same training cohort and validation cohort but validated the applicability of our diagnostic models in a population with a risk of both HCC and metastases. Finally, this study used retrospective multicenter data to validate the models, and a future prospective study is required for further validation of the current recommendations.

## Conclusions

In addition to rim enhancement, unclear borders and washout type II were defined as reliable features for differentiating HCC from LM. Both random forest and generalized linear models had higher sensitivity and accuracy than LI-RADS in the differentiation of HCC from LM in patients with chronic hepatitis and extrahepatic malignancy.

## Electronic supplementary material

Below is the link to the electronic supplementary material.

**Supplementary Material 1**. **Table S1**. The annotation of variables in our study. **Table S2**. Inter-observer agreement of LI-RADS between two senior radiologists. **Table S3**. Inter-observer agreement of arterial phase enhancement patterns between two senior radiologists. **Table S4**. Explore the optimal mild washout time between LM and HCC.

## Data Availability

Data generated or analyzed during the study are available from the corresponding author by request.

## Abbreviations

HCC: Hepatocellular carcinoma
LM: Liver metastasis
CEUS: Contrast-enhanced ultrasound
CEUS LI-RADS: Contrast-Enhanced Ultrasound Liver Imaging Reporting and Data System
GBM: Gradient-boosted model
GLM: Generalized linear model
