# Supplementary material for: Comparison of machine learning models and CEUS LI-RADS in differentiation of hepatic carcinoma and liver metastases in patients at risk of both hepatitis and extrahepatic malignancy
Source: Cancer Imaging. 2023 Jun 19;23:63. doi: 10.1186/s40644-023-00573-8 (PMC10278254; doi:10.1186/s40644-023-00573-8)
Supplement: Supplementary file 1 — Supplementary Material 1. Table S1. The annotation of variables in our study. Table S2. Inter-observer agreement of LI-RADS between two senior radiologists. Table S3. Inter-observer agreement of arterial phase enhancement patterns between two senior radiologists. Table S4. Explore the optimal mild washout time between LM and HCC. [file 40644_2023_573_MOESM1_ESM.docx]

**Supplementary material**

**Table S1. The annotation of variables in our study.**

**Table S2. Inter-observer agreement of LI-RADS between two senior radiologists.**

**Table S3. Inter-observer agreement of arterial phase enhancement patterns between two senior radiologists.**

**Table S4. Explore the optimal mild washout time between LM and HCC.**

**Table S1. The annotation of variables in our study.**

| **Variables** | **Annotation** |
| --- | --- |
| Arterial phase | The arterial phase was defined as 10 to 30-45s seconds after administration. (1) |
| Arterial phase Hyper-enhancement | Arterial phase hyperenhancement is a lesion becoming globally or partially hyperechoic (but not with rim or globular peripheral distribution) compared to the surrounding parenchyma in the arterial phase.(2) |
| Arterial phase Iso-enhancement | Arterial phase iso-enhancement is defined as a lesion becoming globally or partially iso-echoic compared to the surrounding parenchyma in the arterial phase. (2) |
| Arterial phase Hypo-enhancement | Arterial phase hypo-enhancement is defined as a globally or partially hypo-echoic lesion compared to the surrounding parenchyma in the arterial phase. (2) |
| Heterogeneity | Heterogeneous enhancement by two or more enhancements echoes in the arterial phase. (3) |
| Washout | Washout is when the lesion becomes hypoechoic compared to the surrounding parenchyma in the portal-venous phase. (2) |
| Mild washout | Washout appeared before 60 s following contrast injection.(2) |
| Marked washout | The lesion became markedly hypo-enhanced or punched out (otherwise defined as ‘‘mild”) within two minutes.(2) |
| Washout type II | Washout appeared before 54 s following contrast injection and combined with marked washout, defined as “Washout type II”. |
| Unclear Border | An unclear border on CUES was defined as a nodular-shaped tumor with a burr or fuzzy margin on all axial, coronary, and sagittal imaging. (3) |
| Tumor artery | Vasculature in tumors was assessed in the arterial phase using CEUS. (3) |
| Wheel enhancement | Wheel enhancement in tumors was assessed in the arterial phase using CEUS. (4) |
| Rim enhancement | Ring enhancement in tumors was assessed in the arterial phase using CEUS. (2) |
| Jaundice | Jaundice is a condition in which the skin, whites of the eyes and mucous membranes turn yellow because of a high level of bilirubin, a yellow-orange bile pigment. |
| Abdomen pain | Abdominal pain caused by the different causes. |
| Vascular invasion | Postoperative pathology showed that the tumor invaded the blood vessels. |
| Hepatitis | Hepatitis is commonly defined as hepatitis virus infection (A~E). Other causes include heavy alcohol use, autoimmune diseases and non-alcoholic steatohepatitis. (5-7) |
| Diameter of liver cancer | The maximal diameter of colorectal liver metastases was measured by ultrasound or other imaging methods, recording the average diameter of three maximal diameter (a, b, c). (8) |

**References:**

1. Zhou H, Zhang C, Du L, et al. Contrast-Enhanced Ultrasound Liver Imaging Reporting and Data System in Diagnosing Hepatocellular Carcinoma: Diagnostic Performance and Interobserver Agreement. Ultraschall Med. 2022;43(1):64-71.

2. Terzi E, Iavarone M, Pompili M, et al. Contrast ultrasound LI-RADS LR-5 identifies hepatocellular carcinoma in cirrhosis in a multicenter restropective study of 1,006 nodules. J Hepatol. 2018;68(3):485-92.

3. Zheng W, Li Q, Zou XB, et al. Evaluation of Contrast-enhanced US LI-RADS version 2017: Application on 2020 Liver Nodules in Patients with Hepatitis B Infection. Radiology. 2020;294(2):299-307.

4. Dietrich CF, Dong Y, Kono Y, et al. LI-RADS ancillary features on contrast-enhanced ultrasonography. Ultrasonography. 2020;39(3):221-8.

5. Razavi H. Global Epidemiology of Viral Hepatitis. Gastroenterol Clin North Am. 2020;49(2):179-89.

6. Autoimmune hepatitis. Nat Rev Dis Primers. 2018;4:18018.

7. Sehrawat TS, Liu M, Shah VH. The knowns and unknowns of treatment for alcoholic hepatitis. Lancet Gastroenterol Hepatol. 2020;5(5):494-506.

8. Han K, Kim JH, Yang SG, et al. A Single-Center Retrospective Analysis of Periprocedural Variables Affecting Local Tumor Progression after Radiofrequency Ablation of Colorectal Cancer Liver Metastases. Radiology. 2021;298(1):212-8.

**Table S2. Inter-observer agreement of LI-RADS between two senior radiologists.**

|  | **Radiologist 1 review: No. (%)** | | | | | |  |
| --- | --- | --- | --- | --- | --- | --- | --- |
| **Radiologist 2 review: No. (%)** |  | LI-RADS.3 | LI-RADS.4 | LI-RADS.5 | LI-RADS.M | Total |  |
|  | LI-RADS.3 | 14 | 3 | 0 | 0 | 17 | |
|  | LI-RADS.4 | 0 | 31 | 8 | 3 | 42 | |
|  | LI-RADS.5 | 0 | 1 | 135 | 14 | 150 | |
|  | LI-RADS.M | 0 | 0 | 23 | 232 | 255 | |
|  | Total | 14 | 35 | 166 | 249 | 464 | |
| Kappa value: 0.845, 95% CI: 0.803 - 0.888, SE: 0.0215 | | | | | | |  |

**Table S3. Inter-observer agreement of arterial phase enhancement patterns between two senior radiologists.**

|  | **Radiologist 1 review: No. (%)** | | | | |
| --- | --- | --- | --- | --- | --- |
| **Radiologist 2 review: No. (%)** |  | Hypo-enhancement | Iso-enhancement | Hyper-enhancement | Total |
|  | Hypo-enhancement | 71 | 0 | 3 | 74 |
|  | Iso-enhancement | 5 | 21 | 14 | 40 |
|  | Hyper-enhancement | 2 | 16 | 332 | 350 |
|  | Total | 78 | 37 | 349 | 464 |
| Kappa value: 0.850, 95% CI: 0.802 – 0.898, SE: 0.0245 | | | | | |

**Table S4. Explore the optimal mild washout time between HCC and LM.**

| **Parameter** | **HCC** | **LM** | **Standardize difference** | **Accuracy** |
| --- | --- | --- | --- | --- |
| WASHOUT.TIME categorical (1) |  |  | 1.16 (0.89, 1.42) | 70.2 |
| ≤68 | 69 (39.66%) | 86 (87.76%) |  |  |
| >68 | 105 (60.34%) | 12 (12.24%) |  |  |
| WASHOUT.TIME categorical (2) |  |  | 1.22 (0.95, 1.49) | 71.7 |
| ≤67 | 65 (37.36%) | 86 (87.76%) |  |  |
| >67 | 109 (62.64%) | 12 (12.24%) |  |  |
| WASHOUT.TIME categorical (3) |  |  | 1.24 (0.97, 1.50) | 72.1 |
| ≤66 | 64 (36.78%) | 86 (87.76%) |  |  |
| >66 | 110 (63.22%) | 12 (12.24%) |  |  |
| WASHOUT.TIME categorical (4) |  |  | 1.27 (1.00, 1.54) | 72.8 |
| ≤65 | 62 (35.63%) | 86 (87.76%) |  |  |
| >65 | 112 (64.37%) | 12 (12.24%) |  |  |
| WASHOUT.TIME categorical (5) |  |  | 1.45 (1.17, 1.73) | 76.8 |
| ≤60 | 50 (28.74%) | 85 (86.73%) |  |  |
| >60 | 124 (71.26%) | 13 (13.27%) |  |  |
| WASHOUT.TIME categorical (6) |  |  | 1.47 (1.19, 1.75) | 77.2 |
| ≤58 | 49 (28.16%) | 85 (86.73%) |  |  |
| >58 | 125 (71.84%) | 13 (13.27%) |  |  |
| WASHOUT.TIME categorical (7) |  |  | 1.47 (1.19, 1.75) | 77.2 |
| ≤57 | 49 (28.16%) | 85 (86.73%) |  |  |
| >57 | 125 (71.84%) | 13 (13.27%) |  |  |
| WASHOUT.TIME categorical (8) |  |  | 1.45 (1.17, 1.72) | 77.2 |
| ≤56 | 48 (27.59%) | 84 (85.71%) |  |  |
| >56 | 126 (72.41%) | 14 (14.29%) |  |  |
| WASHOUT.TIME categorical (9) |  |  | 1.49 (1.21, 1.77) | 77.9 |
| ≤55 | 46 (26.44%) | 84 (85.71%) |  |  |
| >55 | 128 (73.56%) | 14 (14.29%) |  |  |
| WASHOUT.TIME categorical (10) |  |  | 1.55 (1.27, 1.83) | 79.5 |
| ≤54 | 41 (23.56%) | 83 (84.69%) |  |  |
| >54 | 133 (76.44%) | 15 (15.31%) |  |  |
| WASHOUT.TIME categorical (11) |  |  | 1.47 (1.19, 1.75) | 78.6 |
| ≤53 | 41 (23.56%) | 81 (82.65%) |  |  |
| >53 | 133 (76.44%) | 17 (17.35%) |  |  |

HCC: Hepatocellular carcinoma; LM: Liver metastasis;
